# HNC0014, a Multi-Targeted Small-Molecule, Inhibits Head and Neck Squamous Cell Carcinoma by Suppressing c-Met/STAT3/CD44/PD-L1 Oncoimmune Signature and Eliciting Antitumor Immune Responses

**DOI:** 10.3390/cancers12123759

**Published:** 2020-12-14

**Authors:** Jih-Chin Lee, Alexander T.H. Wu, Jia-Hong Chen, Wen-Yen Huang, Bashir Lawal, Ntlotlang Mokgautsi, Hsu-Shan Huang, Ching-Liang Ho

**Affiliations:** 1 Department of Otolaryngology-Head and Neck Surgery, Tri-Service General Hospital, National Defense Medical Center, 325 Cheng-Kung Road Section 2, Taipei 114, Taiwan; doc30450@gmail.com; 2 The Ph.D. Program of Translational Medicine, College of Science and Technology, Taipei Medical University, Taipei 11031, Taiwan; chaw1211@tmu.edu.tw; 3 Graduate Institute of Medical Sciences, National Defense Medical Center, Taipei 114, Taiwan; 4 Clinical Research Center, Taipei Medical University Hospital, Taipei Medical University, Taipei 11031, Taiwan; 5 Graduate Institute of Clinical Medicine, College of Medicine, Taipei Medical University, Taipei 11031, Taiwan; ndmc_tw.tw@yahoo.com.tw; 6 Division of Hematology/Oncology, Department of Medicine, Tri-Service General Hospital, National Defense Medical Center, Taipei 114, Taiwan; 7 Department of Radiation Oncology, Tri-Service General Hospital, National Defense Medical Center, Taipei 114, Taiwan; hwyyi@yahoo.com.tw; 8 Ph.D. Program for Cancer Molecular Biology and Drug Discovery, College of Medical Science and Technology, Taipei Medical University, Taipei 11031, Taiwan; bashirlawal12@gmail.com (B.L.); d621108006@tmu.edu.tw (N.M.); 9 Graduate Institute for Cancer Biology & Drug Discovery, College of Medical Science and Technology, Taipei Medical University, Taipei 11031, Taiwan; 10 Ph.D. Program in Biotechnology Research and Development, College of Pharmacy, Taipei Medical University, Taipei 11031, Taiwan; 11 Division of Hematology and Oncology Medicine, Department of Internal Medicine, Tri-Service General Hospital, National Defense Medical Center, Taipei 114, Taiwan

**Keywords:** head and neck squamous carcinoma (HNSCC), cisplatin resistance, tumorsphere-derived exosomes (Exo^sp^), cancer-associated fibroblast (CAF), c-Met/STAT3/CD44/PD-L1 signaling, small-molecule immunotherapeutic drug

## Abstract

**Simple Summary:**

Cancer stem cells (CSCs) in head and neck squamous cell carcinoma (HNSCC) possess unlimited self-renewal capacity, resist treatments and induce tumor repopulation after interventions. Here, we observed HNSCC CSCs secreted exosomes containing c-Met, STAT3 (also the phosphorylated form of c-Met and STAT3), CD44, and PD-L1 oncogenic signaling molecules. CSC-derived exosomes, in part, transform fibroblasts (NFs) into cancer-associated fibroblasts (CAFs), establish drug resistance, and an immune-evasive tumor microenvironment (TME). We demonstrated HNC0014, a novel small-molecule drug, suppresses HNSCC tumorigenesis, CSC generation and prevents CAF transformation by decreasing the aforementioned oncogenic signaling molecules’ expression in both HNSCC cells and CSC-derived exosomes.

**Abstract:**

Despite advancements in diagnostic and standard treatment modalities, including surgery, radiotherapy, and chemotherapy, overall survival rates of advanced-stage head and neck squamous cell carcinoma (HNSCC) patients have remained stagnant for over three decades. Failure of these treatment modalities, coupled with post-therapy complications, underscores the need for alternative interventions and an in-depth understanding of the complex signaling networks involved in developing treatment resistance. Using bioinformatics tools, we identified an increased expression of c-Met, STAT3, and CD44 corresponding to a poor prognosis and malignant phenotype of HNSCC. Subsequently, we showed that tumorsphere-derived exosomes promoted cisplatin (CDDP) resistance and colony and tumorsphere formation in parental HNSCC cells, accompanied by an increased level of oncogenic/immune evasive markers, namely, c-Met, STAT3, CD44, and PD-L1. We then evaluated the therapeutic potential of a new small molecule, HNC0014. The molecular docking analysis suggested strong interactions between HNC0014 and oncogenic molecules; c-Met, STAT3, CD44, and PD-L1. Subsequently, we demonstrated that HNC0014 treatment suppressed HNSCC tumorigenic and expression of stemness markers; HNC0014 also reduced cancer-associated fibroblast (CAF) transformation by Exo^sp^- and CAF-induced tumorigenic properties. HNC0014 treatment alone suppressed tumor growth in a cisplatin-resistant (SAS tumorspheres) mouse xenograft model and with higher inhibitory efficacy when combined with CDDP. More importantly, HNC0014 treatment significantly delayed tumor growth in a syngeneic mouse HNSCC model, elicited an antitumor immune profile, and reduced the total c-Met, STAT3, and their phosphorylated forms, PD-L1 and CD44, contents in serum exosomes. Collectively, our findings provide supports for HNC0014 as a multi-targeted immunotherapeutic lead compound for further development.

## 1. Introduction

Cancer is the second leading cause of global mortality, with a current global prevalence of 18.1 million [1], and it is estimated to scale up by 75% to approximately 25 million cases before 2035 [2]. Asia, Africa, and South and Central America account for more than 60% of new global cancer cases and 70% of deaths [3]. It is anticipated that less developed countries will experience an 80% increase in cancer-related deaths by 2025 [4]. Head and neck cancers (HNCs) are a heterogeneous metabolic and genetic collection of malignancies of the pharynx, oral cavity, paranasal sinuses, lips, salivary glands, esophagus, and larynx [5], which account for about 4.9% of all known cancer sites [6]. HNCs are the seventh most often occurring and ninth most fatal cancers [7], with 5-year survival rates ranging 12–93% [5]. In 2020, there were more than 84,000 new HNC cases and more than 30,000 deaths in the United States alone [5], and approximately 90% of HNC cells are pathologically squamous carcinomas (HNSCCs). Annual diagnoses were estimated to increase to about 833,000 new cases in 2020 [8]. However, in South Asian countries, tobacco use, areca nut chewing, and excessive alcohol consumption account for about 75% of cases of HNSCC [9], presenting a regional medical unmet need.

The epidermal growth factor receptor (EGFR), a member of the ErbB family of receptor tyrosine kinases, is overexpressed, mutated, or abnormally activated in about 80–100% of HNSCC cases [3]. EGFR downstream signaling includes phosphatidylinositol 3-kinase (PI3K)/Akt/mammalian target of rapamycin (mTOR), mitogen-activated protein kinase (MAPK), and signal transducer and activator of transcription 3 (STAT3), all of which lead to cellular proliferation, motility, adhesion, differentiation, survival, and metastasis [10,11]. Accumulating evidence indicates that besides EGFR activation, the mesenchymal-to-epithelial transition factor (c-Met) and its ligand, hepatocyte growth factor (HGF), are also aberrantly activated in HNSCC. They are known to cross-talk with the EGFR and vascular endothelial growth factor receptor (VEGFR) to promote cell invasion, proliferation, angiogenesis, metastasis, and drug resistance [12]. These findings strongly suggest the need for multi-targeted therapeutic drugs.

Mono-treatment modalities, either radiation or surgery alone, provide satisfactory outcomes in about 30% of early-stage HNSCC patients, while about 66% of patients advance to stage III and IV disease [8]. Management of advanced HNSCC involves multiple treatment modalities such as surgery, chemotherapy, radiotherapy, and immunotherapy [13]. However, despite advancements in diagnoses and effective treatment modalities, such as intensity-modulated radiotherapy (IMRT), low-invasive transoral surgery, immunotherapy (anti-programmed death-ligand 1 (PD-L1) therapy), and drug targeting (anti-EGFR therapy) [5], overall survival (OS) rates of advanced-stage HNSCC patients have remained poor [8]. Lamentably, more than half of patients develop recurrence in distant or nearby sites within 24 months of treatment [14,15]. Failure of surgical approaches and resistance to adjuvant radiotherapy and chemotherapy [16], coupled with post-therapy complications such as renal and mucosal impairments, necrosis, immune suppression, muscle fibrosis, and mandibular fractures, severely compromise life quality and lead to high morbidity in HNC patients [14,17].

Currently, standard second-line chemotherapy for the treatment of metastatic and recurrent HNSCC is lacking. Although HNSCC patients initially respond to cetuximab, an FDA-approved EGFR inhibitor, the efficacy remains unsatisfactory owing to the HGF/c-Met aberrant signaling [18,19]. Analysis of clinical trial results using monotherapy with immune-checkpoint inhibitors also revealed limited efficacy compared to standard chemotherapy. Other mono-targeted approaches using inhibitors of STAT3, mTOR, and NF-ĸB also showed limited efficacy [20,21], thereby underscoring the need for new approaches to overcome current challenges.

Preclinical studies have shown that using a combination of different mono-therapeutic agents with different mechanisms of action, such as EGFR inhibitors, cannot overcome the current challenges of drug resistance in HNSCC. Therefore, multipronged approaches involving targeting tumor cells, the tumor microenvironment, and immune evasion are a way forward. Based on these premises, we evaluated a new small-molecule drug, HNC0014, based on our previously established TC-N19, which was shown to suppress both EGFR- and c-Met-associated signaling in non-small lung cancer [22]. Herein, we provide in vitro and in vivo evidence that HNC0014 exhibits anti-HNSCC effects via inhibiting tumorigenic/stemness signature c-Met/STAT3/CD44/PD-L1, reducing cancer-associated fibroblast (CAF) transformation, and eliciting antitumor immune profiles.

## 2. Materials and Methods

### 2.1. Cell Culture and Reagents

SAS and HSC-3 cell lines were obtained from the Japanese Collection of Research Bioresources (JCRB) Cell Bank. Normal human gingival fibroblasts were purchased from the American Type Culture Collection (ATCC). All cells were maintained according to recommended culture conditions. Cisplatin (CDDP) was purchased from SelleckChem (Hsinchu, Taiwan), while HNC0014 was synthesized as described in our previous study [23]. Stock solutions of HNC0014 (10 mM) and cisplatin (10 mM) were prepared in dimethyl sulfoxide (DMSO; Sigma Aldrich, St. Louis, MO, USA) and stored at −20 °C.

### 2.2. Molecular Docking

The ligand (HNC0014) chemical structure was prepared for docking using Avogadro software, while that of the standard c-Met inhibitor (crizotinib) was retrieved in MOL file format from the PubChem database. Both files were converted into PDB format using Pymol and converted into PDBQT format using AutoDock Tools 1.5.6. All protein targets (receptors), including c-MET (PDB ID: 4GG5), PD-L1 (PDB ID: 5JDR), CD44 (PDB:ID1UUH), and STAT3 (PDB ID: 4ZIA), were retrieved as PDB format files from the Protein Data Bank and subsequently converted to PDBQT format. Ligands were prepared for docking by deleting H_2_O molecules, adjusting polar hydrogen, and adding Kollman charges. Molecular docking was performed using AutoDock Vina with all parameters set to default values, and all bonds in the ligand rotated freely while considering the receptor to be rigid. A grid box of 40 × 40 × 40 Å was generated on defined binding site residues of the ligand. The docked ligand–receptor complex was visualized and analyzed using Pymol.

### 2.3. Generation of HNSCC Tumorspheres (Sp)

The parental or Exo^sp^-cultured SAS and HSC-3 cells were seeded (5 × 10^5^ cells/well) and allowed to grow for 96 h in 6-well ultra-low attachment plates (Corning, Corning, NY, USA) in a serum-free medium consisting of Ham’s F12/Dulbecco’s modified Eagle medium (DMEM) (1:1), basic fibroblast growth factor (bFGF; 10 ng/mL, Pepro-Tech, Rocky Hill, NJ, USA), human epidermal growth factor (hEGF, 20 ng/mL), 2 μg/mL 0.2% heparin (Sigma, St. Louis, MO, USA), and 1% streptomycin/penicillin (100 U/mL, Hyclone, Logan, UT, USA). Suspended cells with a diameter of >50 μm were considered a tumorsphere and quantified using a Cell3iMager neo (CC-3000, Mitek Lab Co., Ltd. New Taipei City, Taiwan).

### 2.4. Isolation of Tumorsphere-Derived Exosomes (Exo^sp^)

According to the manufacturer’s protocol, Exo^sp^ were isolated from the 96-h cultured SAS and HSC-3 tumorsphere mixed with the total exosome isolation kit (Thermo Fisher Scientific, Taipei, Taiwan) in a 10:1 ratio, and isolated exosomes were confirmed using an exosome-specific antibody, Alix. Isolated exosomes were quantified using the Bradford protein assay. An estimated 850–1000 µg of exosomes was obtained from 15 mL of SAS or HSC-3 tumorsphere culture.

### 2.5. Exosome-Induced Transformation of Fibroblasts

To transform normal human fibroblasts (NFs) to cancer-associated fibroblasts (CAFs), 1 × 10^5^ cells of NFs were cultured with 30 µg Exo^sp^ in each well of a 6-well plate for 48 h, with and without HNC0014. The NFs and resultant Exo^sp^-transformed CAFs were characterized for α-smooth muscle actin (SMA), and vimentin expression using immunofluorescence imaging with the primary antibody of α-SMA (1:100, cat no. 48938) incubated for 1 h and the secondary antibody of anti-mouse immunoglobulin G (H+L) and an F(ab)2 fragment (1:800, AlexaFluor 488 conjugate, #4408) incubated on 6-well chamber slides (Nunc™, Thermo Fisher Scientific, Rochester, NY, USA). Stained cells were mounted using Vectashield mounting medium with 4′,6-diamidino-2-phenylindole (DAPI) for nuclear staining. A Zeiss Axiophot fluorescence microscope and AxioVision Zeiss software (Carl Zeiss, Oberkochen, Germany) were used for capturing and processing the images. The Exo^sp^-transformed CAF (5 × 10^5^ cells) were seeded overnight in a 10 cm dish and treated with 20 (SAS) or 9 µM (HSC-3) of HNC0014 for 48 h. After the treatments, the cell lysates were harvested and analyzed for c-Met, STAT3, CD44, and PD-L1 expression using a Western blot assay.

### 2.6. Enzyme-Linked Immunosorbent Assay (ELISA)

The respective culture media from the NFs and resultant Exo^sp^-transformed CAFs, with or without HNC0014, were characterized for HGF secretions using a quantitative human Hepatocyte Growth Factor (HGF) ELISA Kit (R&D Systems, Minneapolis, MN, USA.)

### 2.7. Cell Viability Assay

An established protocol of sulforhodamine B (SRB) [24] was used to assay the cell viability of SAS and HSC-3 cells under different treatment regimes. SAS or HSC-3 cells (10,000 cells/well) were seeded in each well of a 96-well plate either alone or co-cultured with Exo^sp^, followed by 48 h of different treatment regimens involving HNC0014 (100, 50, 25, 12.5, 6.25, 3.125, 1.5625 µM) and cisplatin (25, 12.5, 6.25, 3.125, 1.56, 0.78, 0.39 µM). Following 48 h of treatment, cells were washed with phosphate-buffered saline (PBS) and fixed for 60 min with cold trichloroacetic acid. Fixed cells were washed with double-distilled (dd)H_2_0 and stained for 30 min with SRB (0.4% in 1% acetic acid). Acetic acid (1%) was used to remove any unbound stain, and the plates were air-dried. The contents of plates were dissolved in a 20 mM Tris-based solution for 15 min under constant agitation. The cell viability was determined from the absorbance measured at 515 nm with a microplate reader (Molecular Devices, Sunnyvale, CA, USA).

### 2.8. Colony Formation Assay

SAS and HSC-3 cells were seeded at 500 cells/well in 6-well plates (Corning) and treated with HNC0014 at 3 (SAS) and 1.5 µM (HSC-3) for 6 days. At the 7th day, the media were removed, and colonies were stained and processed using the established protocols of sulforhodamine B (SRB) [24]. The colonies of treated cells were quantified and compared with the control using a Cell3iMager neo scanner.

### 2.9. Sodium Dodecyl Sulfate Polyacrylamide Gel Electrophoresis (SDS-PAGE) and Western Blotting

Cell lysates from SAS and HSC-3 cell lines, tumorspheres, exosomes, NFs, and resultant Exo^sp^-transformed CAFs with or without treatment were subjected to protein expression analyses using SDS-PAGE with a Mini-Protean III system (Bio-Rad, Taipei, Taiwan) and transferred onto polyvinylidene difluoride membranes using the Trans-Blot Turbo Transfer System (Bio-Rad, Taipei, Taiwan). Membranes were incubated with primary antibodies at 4 °C overnight, followed by a 1-h incubation with the respective secondary antibodies at room temperature. Immunoreaction signals were detected with an enhanced chemiluminescence (ECL) detection kit, and images were captured using the U.V.P. BioDoc-It system (Upland, CA, USA). All uncropped Western blotting images were shown in Figure S1.

### 2.10. Animal Experiments

All animal experiments were conducted in compliance with the regulations set by the Animal Research Ethics Committee of Taipei Medical University (Protocol LAC-2018-0414). We used NOD/SCID mice (BioLASCO, Taipei, Taiwan) to demonstrate the tumor-initiating abilities of Exo^sp^-cultured SAS cells versus parental SAS by subcutaneously injecting 10^4^ cells into the right flank of each NOD/SCID mouse. To evaluate the anti-HNSCC effect of HNC0014, CDDP-resistant SAS tumorspheres (cultured under serum-deprived conditions, 10^6^ cells/injection) were inoculated into NOD/SCID 6-week-old female mice as described above. After the tumor had become palpable (approximately 2 weeks), the mice were randomly assigned into 4 groups (*n* = 5), namely, the vehicle control, which received normal saline; cisplatin (CDDP, 1 mg/kg, i.p. injection, twice a week); HNC0014 only (10 mg/kg, i.p. injection, five times/week); and their combination (CDDP + HNC0014). The tumor volume and body weight (BW) were monitored and measured at 2-week intervals for a period of 8 weeks. Mice were humanely sacrificed via a cervical dislocation method at the end of the experimental period. Tumor samples were harvested and processed for protein expression (c-Met, CD44, β-catenin, STAT3, PARP, and Caspase 3) profiling using western blot analysis. The tumor volume was calculated based on the formula where the tumor volume = length × width^2^/2 (unit cm^3^). For testing HNC0014 as an immunotherapeutic agent, immunocompetent female C57BL/6 mice (6 week old) were implanted with an in-house mouse oral cancer cell line, mORAL1 (1 × 10^6^ cells per injection, subcutaneous), which was established by a collection of 4-nitroquinoline 1-oxide (4NQO)-induced tongue tumor samples obtained by following a previously established protocol [25]. The CDDP treatment arm was replaced by the anti-PD-L1 antibody regimen (100 μg/kg, once/week, i.p). We designed this experiment based on a previous report [26]. Blood samples were collected for immunological profiling and exosome collection.

### 2.11. Data Analysis

All experiments were conducted in triplicates. Student’s *t*-test analysis of the data generated was carried out using GraphPad Prism software (San Diego, CA, USA). Significant differences were considered at * *p* < 0.05, ** *p* < 0.001, and *** *p* < 0.0001.

## 3. Results

### 3.1. An Increased c-Met-Associated Signaling Network in HNSCC Patients Is Associated with Poor Prognosis and Cancer Stemness

**Figure 1:**
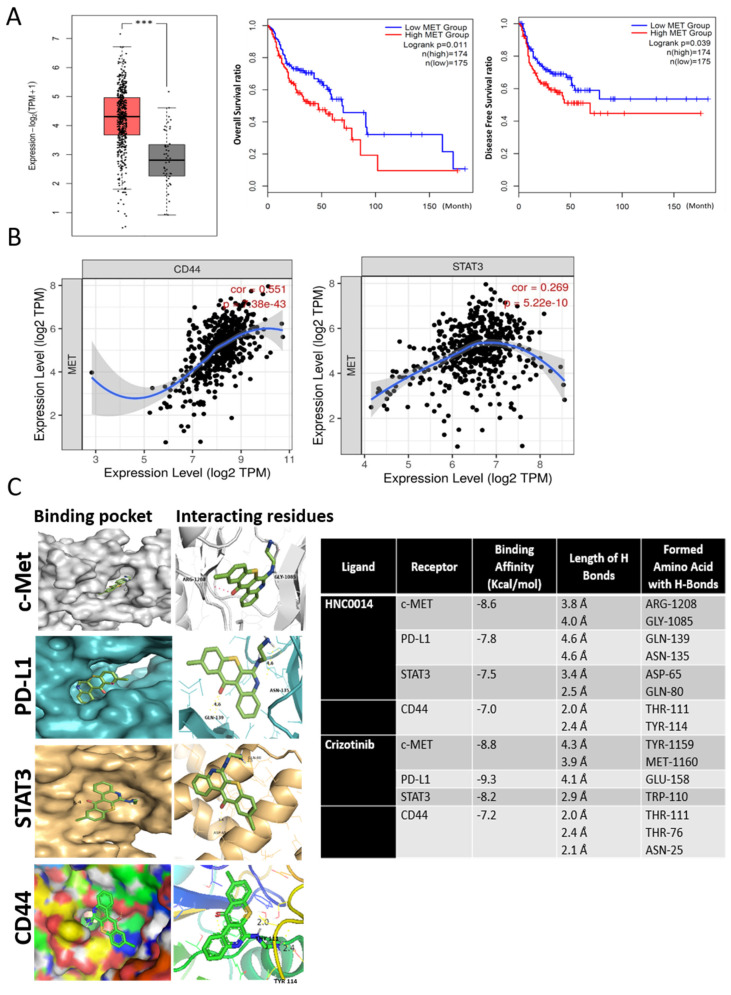
Elevated c-Met/STAT3/CD44 expressions in head and neck squamous cell carcinoma (HNSCC) patients were correlated with a poor prognosis. (**A**) A significantly higher c-Met expression level in patients with head and neck (red box, N = 519) compared to normal counterparts (grey box, N = 44). *P* = 1.0 × 10^−12^. Data analyses from The Cancer Genome Atlas (TCGA) and GTEx datasets (left). Kaplan–Meier plots of the overall survival (middle) and disease-free survival (right) ratios of HNSCC patients. *** *p* < 0.001. (**B**) Correlation analysis of the expression between c-Met and CD44 (left panel, r_s_ = 0.551, *p* = 7.38 × 10^−43^) and c-Met versus STAT3 (right panel, r_s_ = 0.269, *p* = 5.22 × 10^−13^). The expression of target genes was normalized with TBP (TATA-binding protein as an internal control). r_s_ = Spearman’s rank correlation coefficient. (**C**) In silico docking simulations. The receptor-ligand interactions of HNC0014 (green) with c-Met, STAT3, CD44, and PD-L1 are shown. The left panel shows the ligand interaction in the binding pocket of the receptor, while the right panel shows interacting amino acid residues and binding distances. The table (right) indicates the binding energy, the length of H-bonds, and the amino acid residues with H-bonds. Crizotinib, a specific c-Met inhibitor, was used as a positive control for c-Met binding analysis.

Analyses of HNSCC microarrays (GSE31056) and Genotype-Tissue Expression (GTEx) datasets indicated significantly higher c-Met expression levels in patients with HNSCC (left panel, Figure 1A), and this higher c-Met expression predicted significantly lower overall survival (OS, middle panel, Figure 1A) and disease-free survival (DFS, right panel, Figure 1A) rates. Further, we identified a positive correlation between expressions of c-Met and stemness marker CD44 (left panel, Figure 1B) and STAT3 (right panel, Figure 1B). Our bioinformatics data collectively indicated that an elevated c-Met level was associated with elevated levels of STAT3/CD44 and could represent a poor prognostic signature for HNSCC patients.

### 3.2. In Silico Molecular Docking Simulations Predicted Strong Interactions between HNC0014 with c-Met/STAT3/CD44/PD-L1

Following our bioinformatics analysis, we carried out a docking study to determine possible interactions of HNC0014 (green stick) with c-Met, CD44, PD-L1, and STAT3 (Figure 1C). Results generated revealed that the c-Met receptor is the most promising protein target of HNC0014 with the lowest binding energy of −8.6 kcal/mol and binding distance of 3.8 Ă to the ARG-1208 residue and 4.0 Ă to the GLY-1085 residue of the receptor; this is comparable to the binding affinity of −8.8 kcal/mol obtained when c-Met was docked with crizotinib, a specific c-MET inhibitor. Furthermore, HNC0014 interacts with PD-L1, CD44, and STAT3 with respective binding affinities of −7.8, −7.0, and −7.5 kcal/mol (accompanying table). These findings suggest that HNC0014 could be a potent inhibitor of c-Met/STAT3/CD44/PD-L1.

### 3.3. Tumorsphere-Derived Exosomes (Exo^sp^) Promoted c-Met/STAT3/CD44/PD-L1 Expressions, Cisplatin Resistance, and Tumor-Initiating Ability

**Figure 2:**
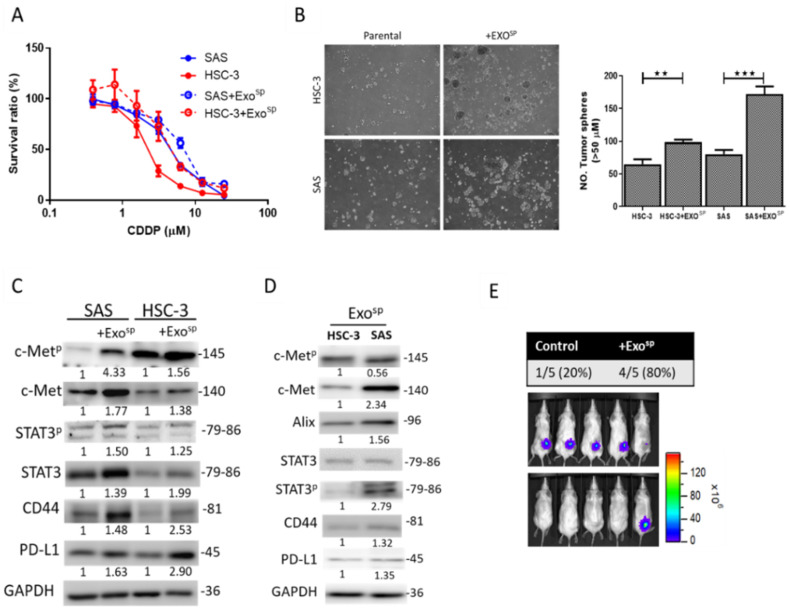
Tumorsphere-derived exosomes (Exo^sp^) promoted the expression of c-Met/STAT3/CD44/PD-L1 expressions, CDDP resistance, and tumor-initiating ability in HNSCC cell lines. SAS and HSC-3 cells cultured with their respective Exo^sp^ showed increased resistance to CDDP (**A**) and enhanced tumor sphere-forming ability (**B**) compared to their parental counterparts. Magnification 4X. (**C**) Western blot showing increased expressions of c-Met-associated signaling components, including c-Met^p^, STAT3, STAT3^p^, CD44, and PD-L1 in both SAS and HSC-3 cells cultured with Exo^sp^. (**D**) Western blots of isolated Exo^sp^ from SAS and HSC-3 cells indicated the presence of STAT3, c-Met, and their phosphorylated forms, PD-L1 and CD44. (**E**) Exo^sp^-transformed SAS cells demonstrated a significantly higher in vivo tumor-initiating ability compared to their parental counterparts. The numbers below the western blots indicate the relative expression ratio, normalized against the internal control (GAPDH). Superscripted P represents the phosphorylated form of c-Met and STAT3. The numbers on the right side of the Western blots indicate the molecular weights of the proteins being examined (in kD). ** *p* < 0.01, *** *p* < 0.001.

Next, we examined the TME-transforming ability of cancer stem cells using tumorspheres as an in vitro surrogate. Exosomes isolated from tumorspheres (Exo^sp^) of both the SAS and HSC-3 cell lines were used to culture parental cancer cells. We found that SAS and HSC-3 cells cultured with their respective Exo^sp^ showed increased tolerance against the chemotherapeutic agent, cisplatin (Figure 2A), and enhanced sphere-forming abilities compared to their parental counterparts (Figure 2B). These tumorigenic properties were correlated with increased expressions of c-Met-associated signaling components, including STAT3^p^, STAT3, CD44, and PD-L1, in both SAS and HSC-3 cells after culturing with Exo^sp^ (Figure 2C). Comparative western blots of isolated Exo^sp^ from both HSC-3 and SAS cell lines contained oncogenic signaling molecules, including STAT3, c-Met, and their phosphorylated forms, PD-L1 and CD44 (Figure 2D). We next examined the tumor-initiating ability of Exo^sp^-cultured SAS cells in vivo. As expected, Exo^sp^-cultured SAS cells demonstrated a significantly higher in vivo tumor-initiating ability compared to the parental counterparts (80% versus 20%, respectively) (Figure 2E).

### 3.4. HNC0014 Treatment Inhibits Tumorigenic and Stemness Properties via Downregulating c-Met/STAT3/PD-L1 Expressions

**Figure 3:**
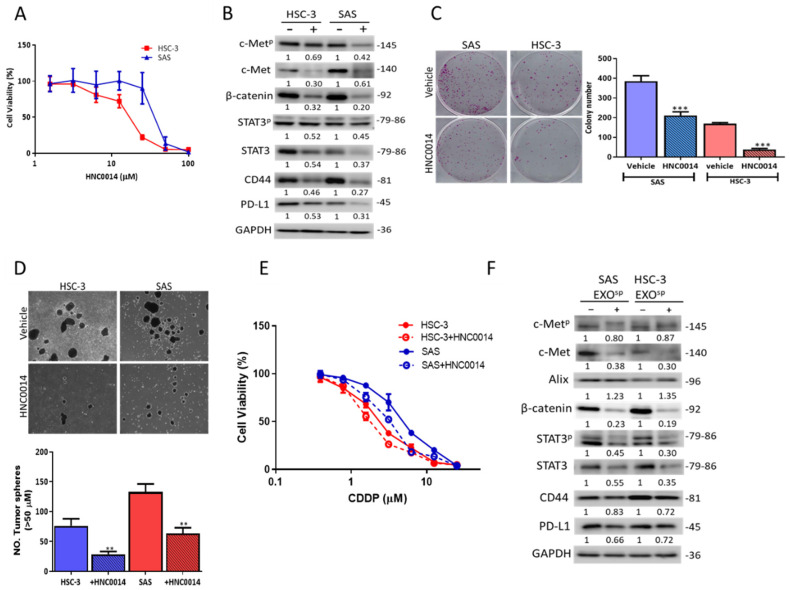
HNC0014 treatment inhibited tumorigenic and stemness properties via downregulating c-Met/STAT/CD44/PD-L1 expression. (**A**) HNC0014 treatment resulted in a dose-dependent reduction in cell viability in both SAS and HSC-3 cells. (**B**) Western blots of HNC0014-treated (+) SAS and HSC-3 cells exhibited lower expression levels of c-Met, c-Met^p^, STAT3, STAT3^p^, PD-L1, CD44, and β-catenin. HNC0014 treatment significantly reduced the colony-forming (**C**) and tumorsphere-forming abilities (**D**) of both SAS and HSC-3 cells. Magnification 4X. (**E**) HNC0014 pretreatment of HSC-3 (2 µM, 24 h) and SAS (5 µM, 24 h) increased the sensitivity of Exo^sp^-transformed HNSCC cells to CDDP treatment. (**F**) HNC0014 treatment significantly reduced the expression of STAT3, STAT3^p^, PD-L1, c-Met, c-Met^p^, CD44, and β-catenin from both exosomes derived from HSC-3 and SAS tumorspheres. ** *p* < 0.01, *** *p* < 0.001.

Subsequently, we assayed the inhibitory activities of HNC0014, a new small molecule modified from TC-N19 [27], against HNSCC cells. First, HNC0014 dose-dependently reduced the cell viability in both SAS and HSC-3 cells (Figure 3A). Western blots of HNC0014-treated SAS and HSC-3 cells exhibited downregulated PD-L1 and total and phosphorylated forms of c-Met and STAT3 expression profiles, along with stemness markers β-catenin and CD44 (Figure 3B). Further, HNC0014-treated cells showed significantly reduced abilities to form colonies (Figure 3C) and tumorspheres (Figure 3D). Furthermore, we demonstrated that HNC0014 pretreatment (5 and 2 µM for SAS and HSC-3, respectively) increased CDDP sensitivity in Exo^sp^-educated HNSCC cells (Figure 3E). Interestingly, the overall abundances of STAT3, STAT3^p^, PD-L1, c-Met, c-Met^p^, and CD44 were markedly lower in exosomes derived from tumorspheres treated with HNC0014 compared to their control counterparts (Figure 3F).

### 3.5. HNC0014 Reduced HNSCC-Induced CAF Transformation and Its Protumorigenic Properties In Vitro

**Figure 4:**
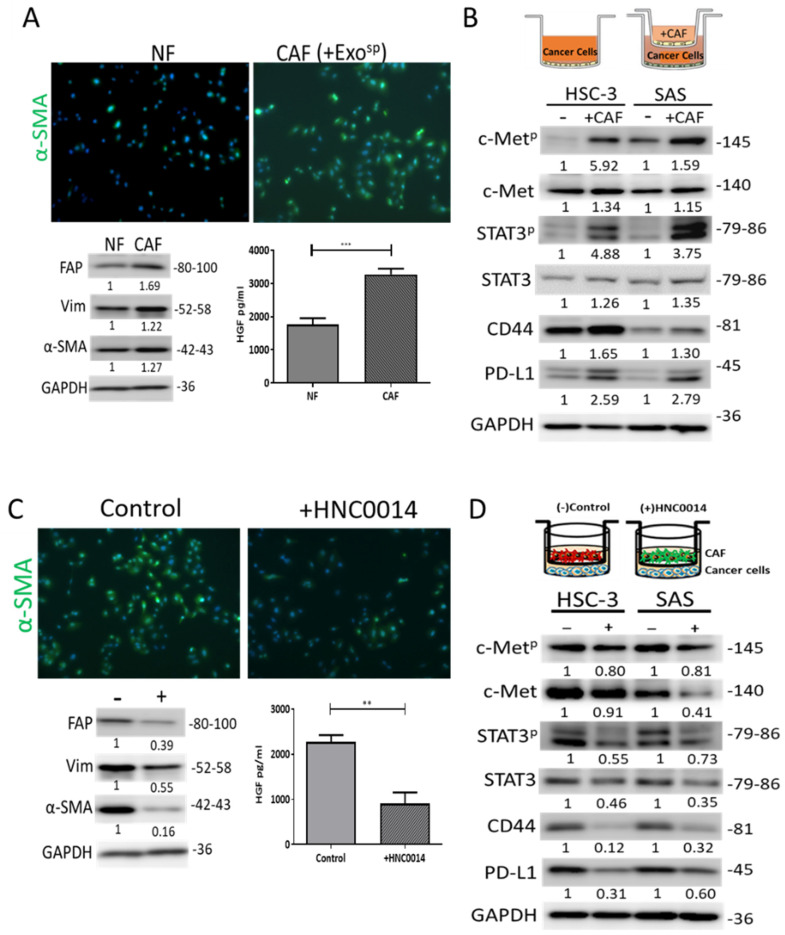
HNC0014 reduced Exo^sp^-induced CAF transformation and its protumorigenic properties in vitro. (**A**) Immunofluorescence images demonstrating the tumorsphere-derived exosome (Exo^sp^) promoted CAF transformation (increased by increased α-SMA green fluorescence). Magnification 20X. Western blots (lower left panel) indicated CAF transformation reflected by the increased expressions of α-SMA, Vim, and FAP. ELISA analysis of Exo^sp^-transformed CAFs (lower right panel) indicated a higher level of hepatocyte growth factor (HGF) secretion than their normal fibroblast (NF) counterparts. (**B**) SAS and HSC-3 cells co-cultured with CAFs showed increased expressions of c-Met/CD44/STAT3/PD-L1 signatures. (**C**) HNC0014 treatment significantly reduced the Exo^sp^-mediated CAF transformation. The expression of α-SMA, Vim, and FAP was reduced in the presence of HNC0014 (lower left panel). ELISA assay showed a reduced HGF secretion by the HNC0014-treated CAF (lower right panel). Magnification 20X. (**D**) Western blots showed a decreased level of c-Met, CD44, STAT3, PD-L1, and phosphorylated forms of c-Met and STAT3 (c-Met^p^ and STAT3^p^ respectively) in SAS and HSC-3 cells co-cultured with CAFs in the presence of HNC0014. ** *p* < 0.01, *** *p* < 0.001.

CAFs were established as one of the major players within the TME involved in the progression of HNCs [28]. Herein, we examined the effects of HNC0014 on CAFs by culturing NFs in the presence of Exo^sp^. Exo^sp^-cultured NFs showed increased α-SMA and vimentin expressions (upper and lower left panels, Figure 4A). Results from our ELISA analysis of the culture medium revealed that Exo^sp^-transformed CAFs secreted a significantly higher level of HGF compared to their NF counterparts (lower right panel, Figure 4A). More importantly, SAS and HSC-3 cells showed increased expressions of c-Met/CD44/STAT3/PD-L1 signatures after being co-cultured with CAFs (Figure 4B). Subsequently, we added HNC0014 to the Exo^sp^-NF culture system and found that HNC0014 treatment reduced CAF transformation (upper and lower left panels, Figure 4C), as evidenced by reduced expressions of α-SMA and vimentin. Consistently, HGF secretion by CAFs was significantly decreased by the addition of HNC0014 (lower right panel, Figure 4C); HNC0014-treated CAFs showed a markedly reduced ability to induce expression of c-Met/CD44/STAT3/PD-L1, and the phosphorylated c-Met/STAT3 signatures in both SAS and HSC-3 cells (Figure 4D).

### 3.6. HNC0014 Treatment Improved Cisplatin Sensitivity and Suppressed Tumorsphere-Initiated Tumor Growth In Vivo

**Figure 5:**
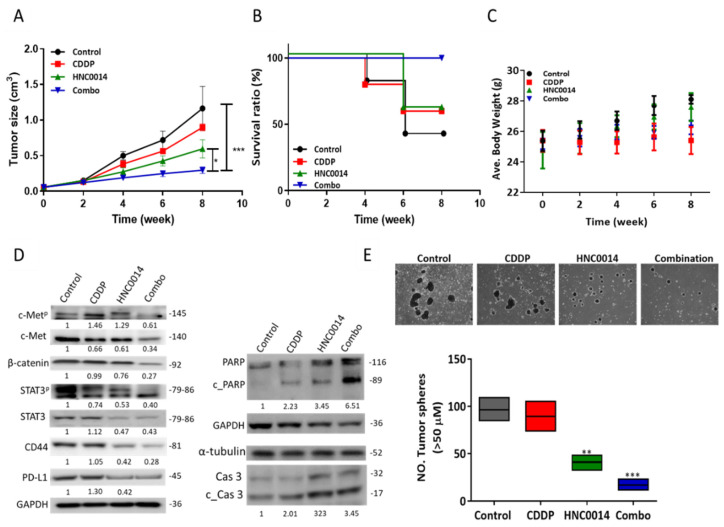
HNC0014 treatment improved cisplatin sensitivity and suppressed tumorsphere-initiated tumor growth in vivo. An SAS tumorsphere-bearing mouse model was established to evaluate HNC0014′s antitumor efficacy. An average tumor volume versus time curve (**A**) shows that HNC0014 treatment significantly delayed tumor growth. The Kaplan–Meier survival curve (**B**) shows a higher overall survival ratio of animals treated with HNC0014 alone or in combination with cisplatin compared to the control counterparts. (**C**) HNC0014 treatment caused no significant changes in the body weights of the animals. (**D**) Western blots from tumor samples of HNC0014 and the combination groups revealed decreased expressions of c-Met/STAT3/CD44/PD-L1 and β-catenin, while increased expression levels of apoptotic markers PARP and caspase-3 (Cas) and their cleaved forms PARP (c_PARP) and Cas3 (c_Cas3), respectively (right panel). (**E**) In vitro tumorsphere-forming assay showed that tumor cells harvested from HNC0014 and the combination groups formed significantly lower numbers of tumorspheres compared to the cisplatin (CDDP) and vehicle control groups. Magnification 4X. ** *p* < 0.01, *** *p* < 0.001.

We further analyzed the anti-HNSCC functions of HNC0014 using an SAS tumorsphere-bearing mouse model. We demonstrated that tumor growth was significantly delayed in mice, which had received HNC0014 treatment (20 mg/kg, five times/week, i.p. injection) compared to the control counterparts (Figure 5A). More importantly, the addition of HNC0014 re-sensitized SAS tumorspheres towards cisplatin treatment, as demonstrated in the significantly reduced tumor burden in the combination arm (Figure 5A). Further, OS ratios of the HNC0014-alone and combination groups were higher than that of the untreated control group (Figure 5B). There was no significant difference in the average body weight among HNC0014 and the combination groups, while a slight decrease was observed in the CDDP-only group (Figure 5 C). Western blots from tumor samples of HNC0014 and the combination groups revealed reduced expression levels of c-Met, c-Met^p^, STAT3, STAT3^p^, CD44, β-catenin, and PD-L1, but increased expressions of the apoptotic markers poly(ADP ribose) polymerase (PARP) and caspase-3 and their cleaved forms (c_PARP and c_Cas 3) (Figure 5D). Furthermore, we cultured harvested tumor cells under serum-deprived conditions. Tumor cells from the HNC0014 and the combined groups formed significantly lower numbers of tumorspheres compared to the CDDP and vehicle counterparts (Figure 5E).

### 3.7. HNC0014 Treatment Suppressed Tumor Growth in a Syngeneic Mouse Model and Exhibited an Antitumor Immune Profile

**Figure 6:**
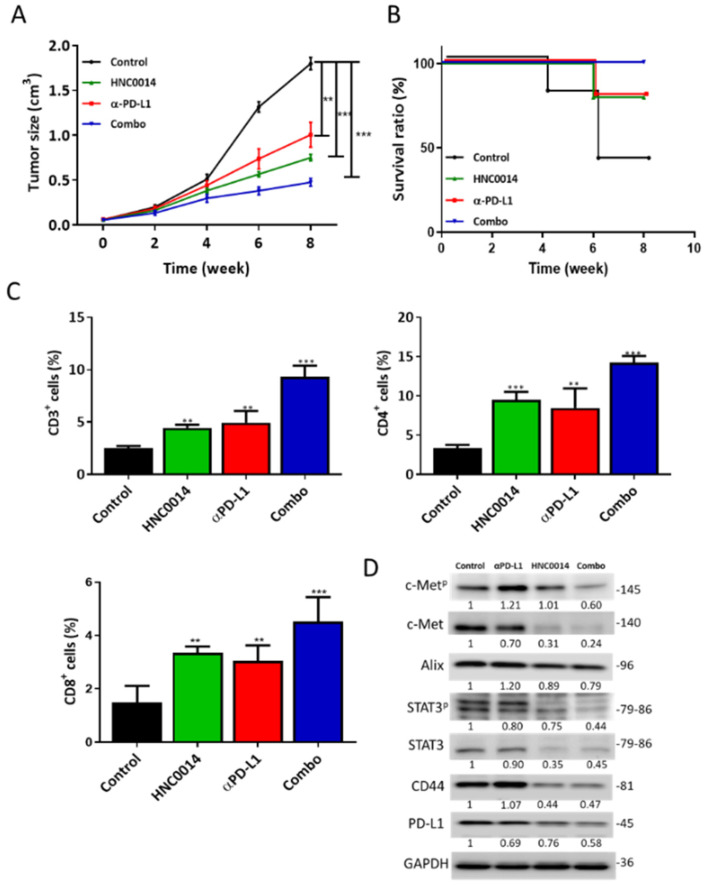
Evaluation of HNC0014 immunotherapeutic functions in syngeneic mouse oral cancer model. (**A**) Tumor volume versus time curve showing slower tumor growth in mice treated with HNC0014 alone, αPD-L1 antibody alone, and the combination of HNC0014 and PD-L1 antibody (αPD-L1), compared to vehicle control. (**B**) Kaplan–Meier survival curve depicting the highest survival ratio of mice in the combined treatment group (HNC0014 + αPD-L1) over the individual treatments, while the control group showed the lowest survival ratio. (**C**) Comparative blood T cell profiles. Flow cytometric results of the blood samples from each group demonstrated significantly higher percentages of CD3+ (total T cells), CD4+ (helper T cells), and CD8+ (cytotoxic T cells) cell populations in all treatment groups, compared to the control counterparts. ** *p* < 0.01, *** *p* < 0.001. (**D**) Western blots of serum exosomes isolated from each group. A markedly reduced expression of c-Met, STAT3, PD-L1, CD44, and the phosphorylated forms of c-Met and STAT3 (c-Met^p^ and STAT3^p^ respectively) in the HNC0014-alone and combination groups were observed compared to the control counterparts. Alix served as an exosomal marker.

After establishing that HNC0014 treatment resulted in the reduced expression of c-Met, STAT3, CD44, β-catenin, and PD-L1, this observation suggested its immune-modulating role. Hence, we used a syngeneic mouse tumor xenograft model to examine this possibility. Tumor growth was significantly delayed in both the HNC0014-alone and anti-PD-L1 antibody (αPD-L1) arms. More importantly, the combination of HNC0014 and αPD-L1 yielded the most significant tumor growth delay (Figure 6A). The combined treatment also resulted in the highest survival ratio, followed by the HNC0014-alone and α-PD-L1 groups, while the control group had the lowest ratio (Figure 6B). Blood samples were collected and profiled. A significantly higher percentage of the CD3+ T cell population was observed in all treatment groups other than the control counterparts (Figure 6C). Similarly, CD4+ and CD8+ T cell populations were significantly higher in HNC0014, αPD-L1, and the combination groups compared to the vehicle control counterparts (Figure 6C). We also harvested the serum exosomes from each group and found that the exosomes from the combination treatment arm contained the lowest expression level of c-Met, STAT3, PD-L1, CD44, and the phosphorylated forms of c-Met and STAT3 (c-Met^p^ and STAT3^p^ respectively) followed by HNC0014-only and αPD-L1(Figure 6D).

## 4. Discussion

This study provided functional and mechanistic evidence of the antitumorigenic properties of HNC0014 against HNSC. We demonstrated, using both in vitro and in vivo models, that HNC0014 exhibited antitumor properties and resulted in an antitumor immune profile via downregulation of c-Met/STAT/CD44/PD-L1 signatures in HNSC. First, we showed that c-Met expression positively correlated with that of STAT3 and CD44 in HNSCC clinical databases, and high c-Met expression predicted significantly lower overall and disease-free survival rates in HNSCC clinical cohorts. In support, a previous study indicated a positive correlation between PD-L1 and c-Met expression in patients with recurrent head and neck cancer [29,30]. Further, c-Met overexpression correlates with the maintenance of cancer stem cell properties in HNSCC and treatment failure [31]. Here, we provided evidence that Exo^sp^ contained c-Met, c-Met^p^, CD44, STAT3, STAT3^p^, and PD-L1. Specifically, Exo^sp^ promoted cisplatin resistance, tumorsphere formation in the parental HNSCC cells, and CAF transformation. In agreement, previous studies demonstrated that exosomes could induce higher expressions of PD-L1, STAT3, β-catenin, CD44, and PI3K/mTOR signaling to promote malignant features of HNSCC [32,33,34]. Accumulating evidence indicated the pivotal roles of cancer-derived exosomes, which promote not only malignant tumor phenotypes but also decrease T cell activation/proliferation by transferring PD-L1 and oncogenic molecules to recipient cells [35,36]. Therefore, our findings that Exo^sp^ contained oncogenic and immune-evasive signaling cargoes added a new layer of complexity to how cancer stem cells transform the tumor microenvironment and promote resistance against conventional and immunotherapeutic agents. Hence, identifying agents that can somehow interrupt tumor-derived, and even CSC-derived, exosomes should lead to improved therapeutic outcomes.

Based on these premises, we evaluated HNS0014, a new small molecule derived from our lead compound TC-N19 [35], a dual EGFR and c-Met inhibitor. In this study, our docking simulations of HNC0014 revealed strong interactions between HNC0014 and c-Met, STAT3, PD-L1, and CD44. Interestingly, HNC0014 interactions with c-Met occurred with much closer proximity to the ligand and a binding affinity of −8.6 kcal/mol compared to the binding affinity (−8.8 kcal/mol) with crizotinib (a clinical c-Met inhibitor). In vitro validation experiments showed HNC0014 mediated various therapeutic effects on parental and Exo^sp^-transformed HNSCC cells’ gene and phenotypic expressions. For instance, we demonstrated that HNC0014 pretreatment re-sensitized Exo^sp^-transformed HNSCC cells to cisplatin and reduced the expression of STAT3, PD-L1, c-Met, CD44, β-catenin, and the phosphorylated forms of c-Met/STAT3. Notably, c-Met and β-catenin signaling promote the expression of PD-L1 on tumor cells, resulting in immune escape [37,38,39]. The observations that HNC0014 treatment led to decreased expression of c-Met and its phosphorylated form (c-Met^p^), CD44, β-catenin, and PD-L1 reinforced our hypothesis that HNC0014 may function as an anti- CSC and immunotherapeutic agent.

Cancer-associated fibroblasts (CAFs) represent one of the most influential stromal cell populations within the TME, which are involved in the progression of HNSCC [28], and targeting them increases chemosensitivity [40]. In agreement with previous studies [41], we reported that Exo^sp^-transformed CAFs expressed higher levels of the specific fibroblast markers FAP, α-SMA, and vimentin, which exhibited higher secretion of HGF compared to their NF counterparts. These markers indicate an activated phenotype that helps maintain the resistance of CAFs to cell death mechanisms and allows them to remain activated [42,43]. According to results generated from a recent clinical study, α-SMA hyperexpression was detected in about 70% of oral cancer patients and positively correlated with an advanced tumor grade, enhanced tumor invasion, and frequent disease relapse, suggesting CAFs’ essential involvement in promoting cancer malignancy and a potential target for intervention [44]. Notably, our data indicated HNC0014 treatment suppressed CAF-mediated enhanced expressions of c-Met/STAT3/CD44/PD-L1 and the phosphorylated forms of c-Met/STAT3 in both SAS and HSC-3 cells in the co-culture system. Considering that stromal cells dominate the HNSCC tumor mass, it is conceivable that drugs such as HNC0014 that can target CAFs may have better treatment effects [45]. The precise mechanisms of action as to how HNC0014 reduced CAF transformation is currently under investigation in our laboratory.

Consistent with the in vitro data, our in vivo study indicated that HNC0014 significantly attenuated the proliferation and overcame CDDP resistance; HNC0014 treatment was also accompanied by reduced c-Met, c-Met^p^, CD44, β-catenin, STAT3, and STAT3^p^, while increased tumor apoptosis was evident by higher expressions of PARP, caspase-3, and their cleaved forms (c_PARP and c_Cas 3). These findings supported the antitumorigenic and possibly anti-CSC functions of HNC0014 in vivo. Based on the inhibitory role of HNC0014 on exosome-induced PD-L1 expression, we anticipated an immunotherapeutic effect of this small molecule.

HNC0014 alone significantly delayed the tumor growth and showed even better results when combined with the anti-PD-L1 antibody; this is conceivable since HNC0014 reduced CD44, β-catenin c-Met, and its phosphorylated form (c-Met^p^), all of which are contributors towards immune suppression [38,46,47]. More importantly, we demonstrated that mice that received HNC0014 alone and in combination with the anti-PD-L1 antibody contained a significantly higher population of CD4+ and CD8+ cells compared to their vehicle controls. An increase in helper T cells (CD4+) and cytotoxic CD8+ T cells reflected an antitumor immunity profile. We also observed an increased serum level of IL-2. IL-2 is a crucial mediator of CD4+ T cell differentiation and function and can regulate the effector and memory responses of CD8+ T cells [48]. Collectively, the increased percentage of CD3+, CD4+, and CD8+ T cells strongly suggested the partial restoration of anitumor immunity in mice treated with HNC0014 and the combination regimen.

## 5. Conclusions

In conclusion, we provided evidence for HNC0014-mediated suppression of tumorigenic properties of HNSC by downregulating c-Met/STAT/CD44/PD-L1 and the phosphorylated c-Met/STAT signature. HNC0014 also normalized the tumor microenvironment by reducing CAF transformation, in part, by reducing tumorsphere-derived exosomes cargoes. In vivo mouse xenograft models revealed the dual functions of HNC0014, where it suppressed tumor growth and cancer stemness while partially restoring antitumor immunity. Further investigation of HNC0014 is warranted for its clinical translation.

## Supplementary Materials

The following are available online at https://www.mdpi.com/2072-6694/12/12/3759/s1, Figure S1: Original western blots for Figure 2, Figure 3, Figure 4, Figure 5 and Figure 6, Table S1: Primary antibodies used for western blots.
