# Supplementary material for: HNC0014, a Multi-Targeted Small-Molecule, Inhibits Head and Neck Squamous Cell Carcinoma by Suppressing c-Met/STAT3/CD44/PD-L1 Oncoimmune Signature and Eliciting Antitumor Immune Responses
Source: Cancers (Basel). 2020 Dec 14;12(12):3759. doi: 10.3390/cancers12123759 (PMC7764918; doi:10.3390/cancers12123759)
Supplement: Supplementary file 1 [file cancers-12-03759-s001.zip › cancers-1033918-XML suppl/cancers-1033918-XML supp table.pdf]

Table S1: Primary antibodies and experimental conditions used in this study

	Target	Dilution	Company and Catalog No.	Predicted MW (kDa)	Observed MW (kDa)
01	GAPDH	1:5000	Proteintech, GAPDH Rabbit pAb, 10494-1-AP	36	36
02	c-Met	1:1000	Proteintech, c-Met Rabbit pAb, 19971-1-AP	156	140
03	STAT3	1:1000	Proteintech, STAT3 Rabbit pAb, 10253-1-AP	79-86	79-86~100
04	CD44	1:1000	Invitrogen, CD44 Mouse mAb, MA5-13890	81	81
05	PD-L1	1:1000	Proteintech, PD-L1 Mouse mAb, 66248-1-Ig	33	45-50
06	$STAT3^{P}$	1:1000	Cellsignal, p-STAT3 Rabbit mAb, #9145s	79-86	79-86
07	Alix	1:1000	Abcam, Alix Rabbit pAb, ab76608	96	96
08	β -catenin	1:1000	Cell Signaling, β –Catenin (6B3) Rabbit mAb , #9582P	92	100
09	Vimentin	1:1000	Cloud Clone, Vimentin Rabbit pAb, PAB040Hu01	52-58	52-58
10	α-SMA	1:1000	Proteintech, α-SMA Rabbit pAb, 14395-1-AP	43	43
11	FAP	1:1000	MYBioSource, FAP alpha (MBS8244119) Rabbit pAb, MBS8241750	80-100	80-100
12	PARP	1:1000	CellSignaling, PARP (46D11) Rabbit mAb #9532	116, 89	116, 89
13	Caspase 3	1:1000	Abcam, Recombinant Anti-Caspase-3 Rabbit mAb, ab184787	32	17-32
14	$c\text{-}Met^{P}$	1:1000	Cell signaling, Phospho-Met Rabbit mAb, 3077S	145	145
15	α-tubulin	1:1000	Abcam, Anti-alpha Tubulin antibody(ab185067)	50	52
Mouse antibodies					
**01**	C-MET	1:1000	abcam, c-Met rabbit mAb, ab51067	156	160
02	CD44	1:1000	abcam, CD44 rabbit mAb, ab189524	82	82
03	PD-L1	1:1000	abcam, PD-L1 rabbit mAb, ab213480	33	40-60

04	STAT3^{p}	1:1000	abcam, p-STAT3 rabbit mAb, ab76315	88	88
05	Alix	1:1000	abcam, Alix rabbit mAb, ab275377	90-100	96
06	c-Met^{p}	1:1000	Cell signaling, Phospho-Met Rabbit mAb, 3077S	145	145
