# Supplementary figures and images for: HNC0014, a Multi-Targeted Small-Molecule, Inhibits Head and Neck Squamous Cell Carcinoma by Suppressing c-Met/STAT3/CD44/PD-L1 Oncoimmune Signature and Eliciting Antitumor Immune Responses
Source: Cancers (Basel). 2020 Dec 14;12(12):3759. doi: 10.3390/cancers12123759 (PMC7764918; doi:10.3390/cancers12123759)

Figure 2

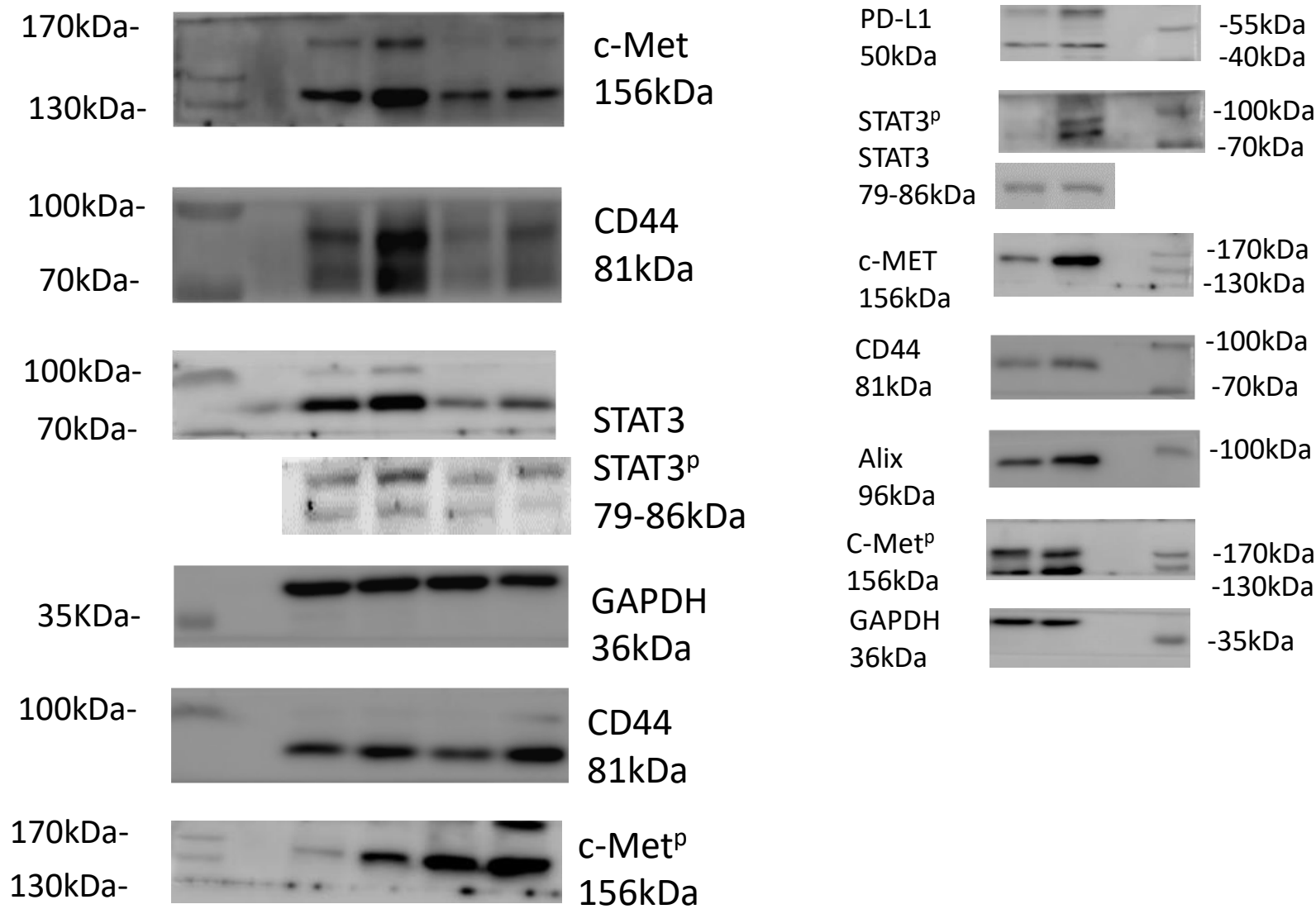

Figure 3

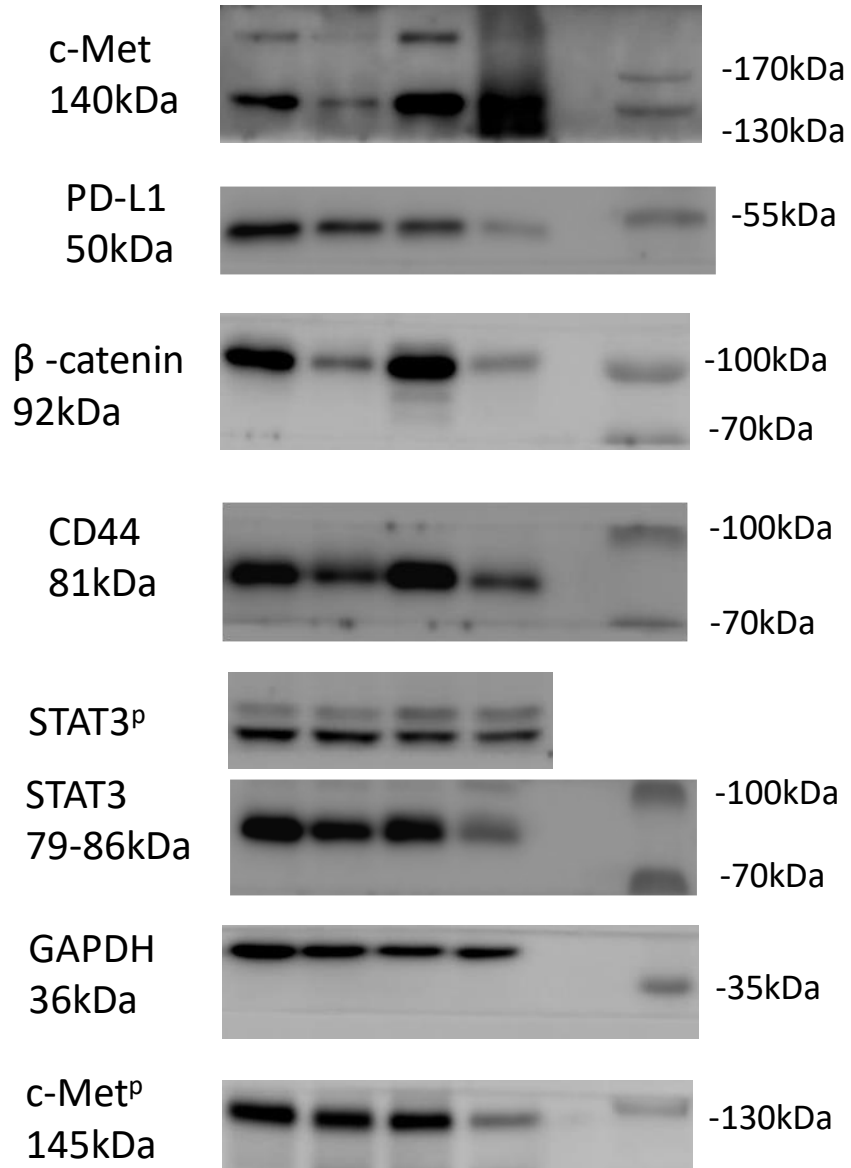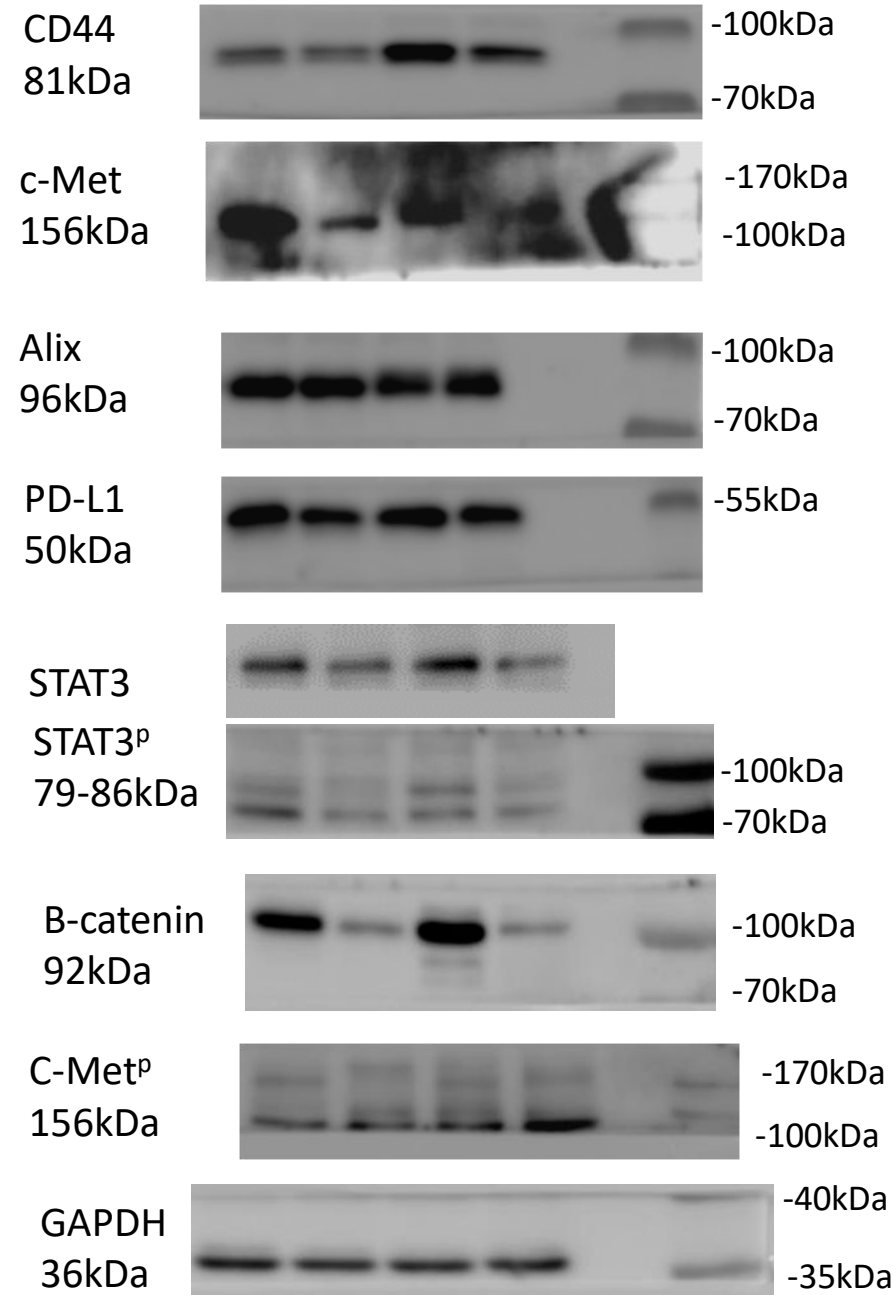

Figure 4

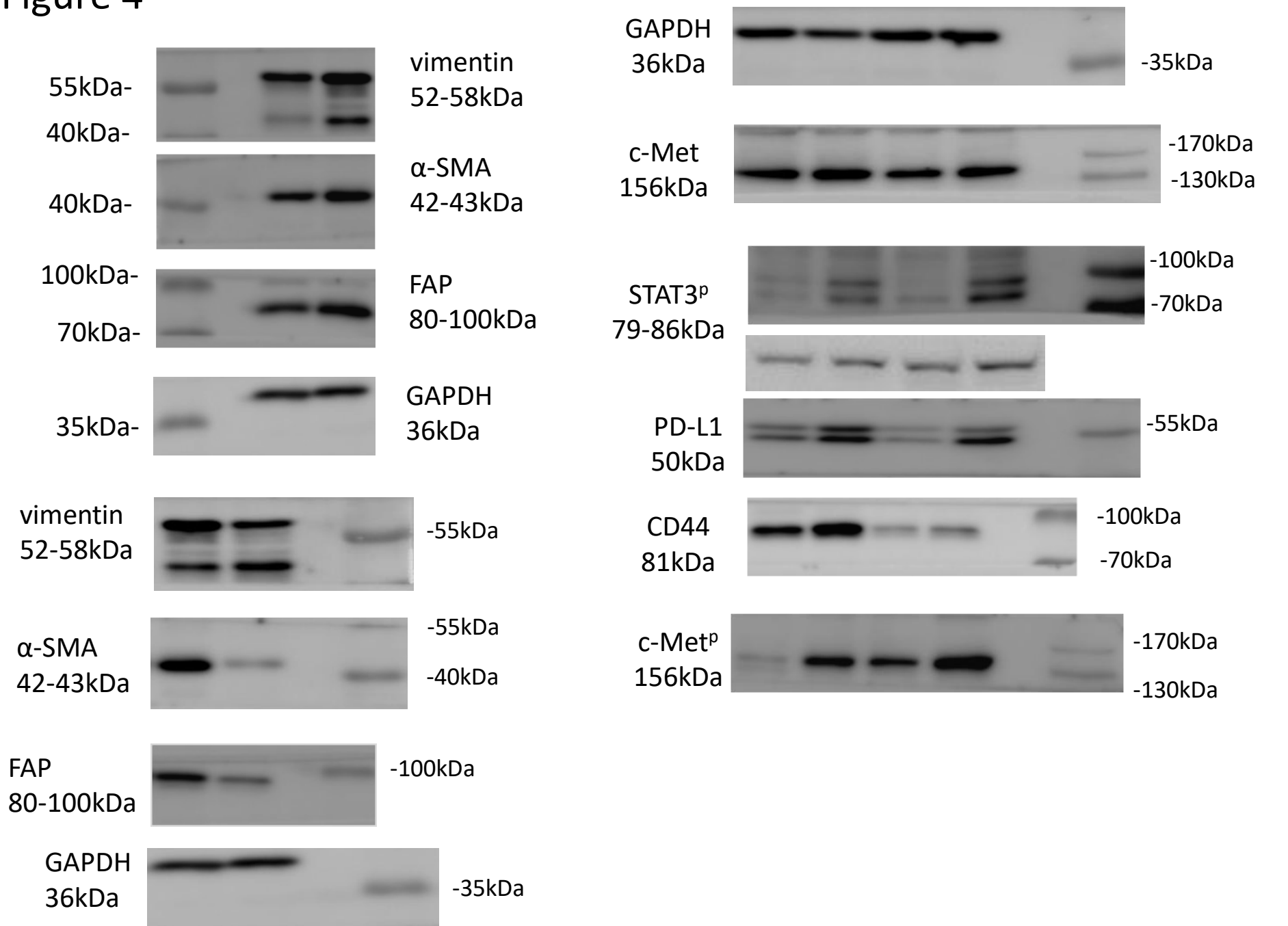

Figure 5

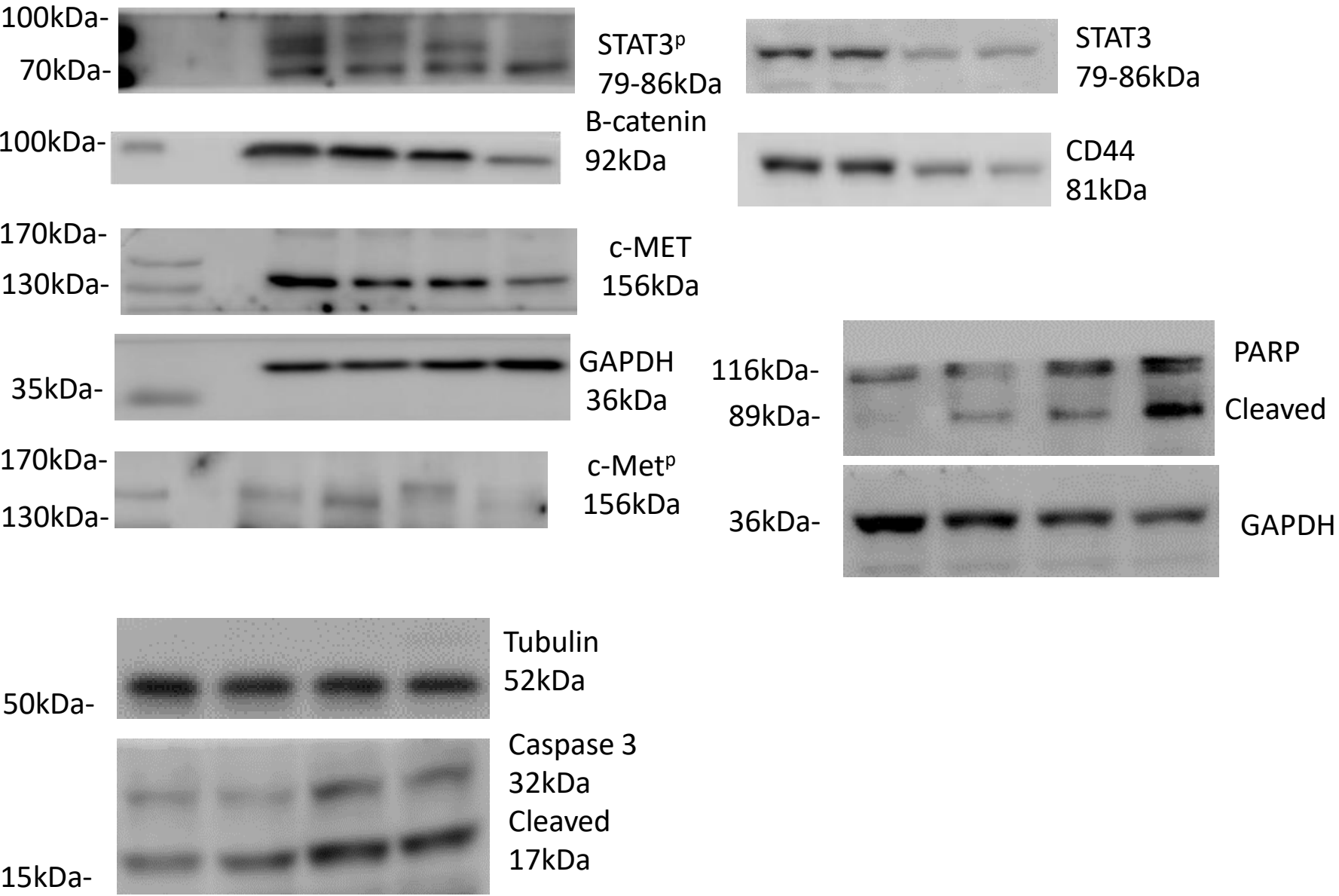

Figure 5 Conti.

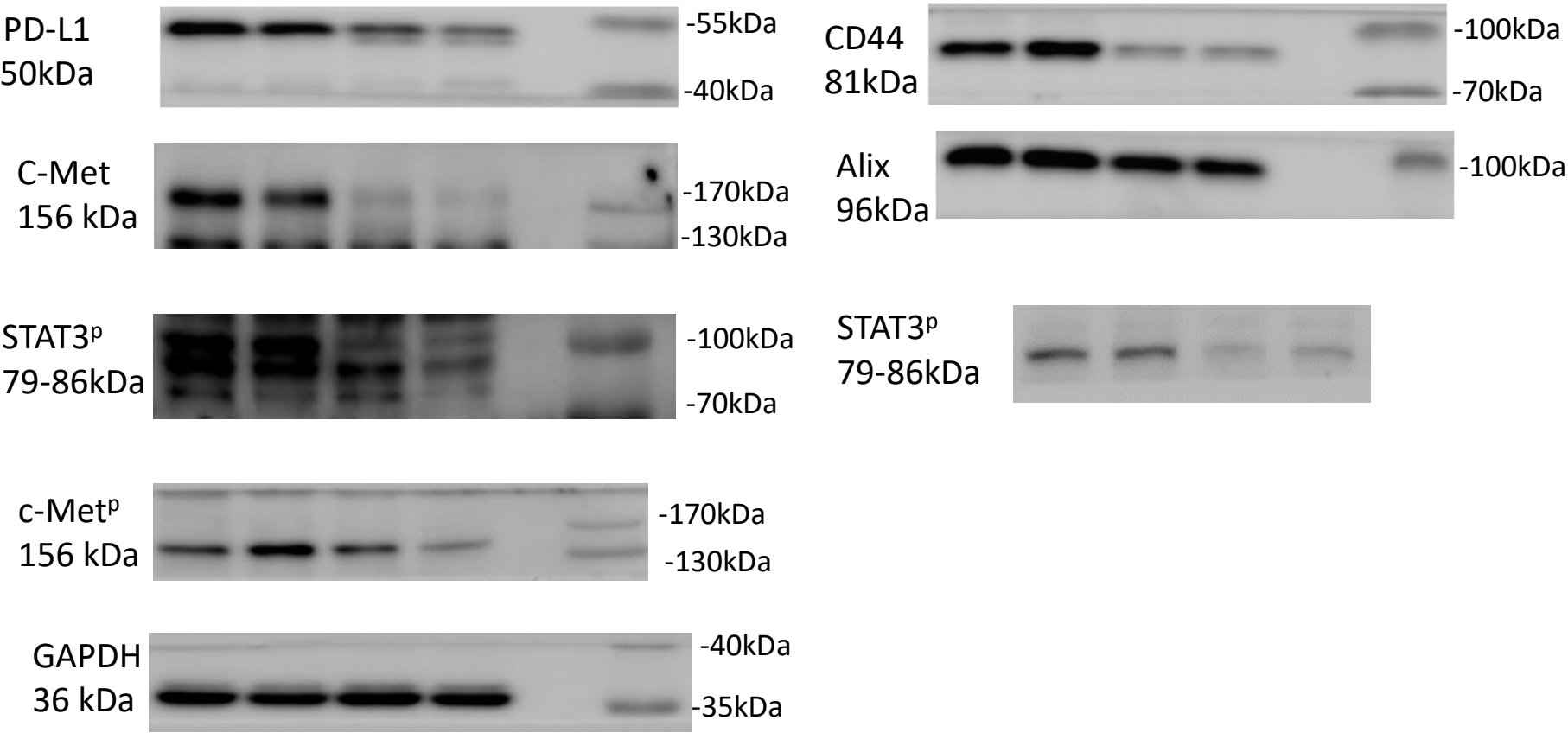

Figure 6

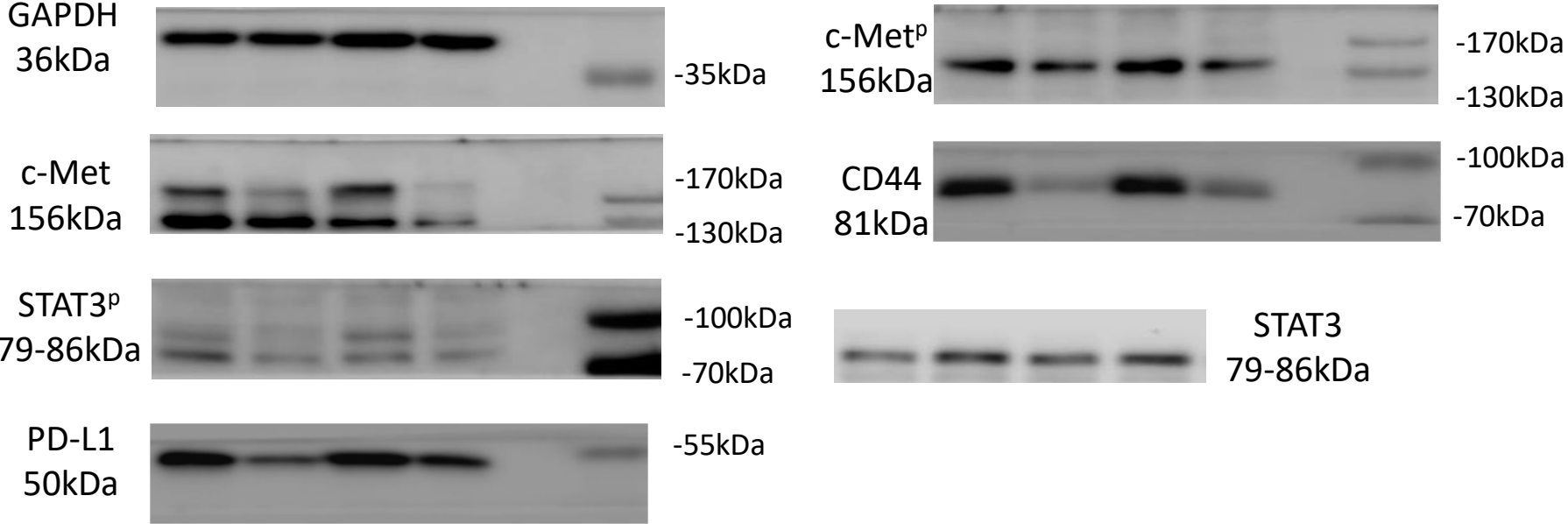

Supplement: Supplementary file 1 [file cancers-12-03759-s001.zip › cancers-1033918-XML suppl/cancers-1033918-XML supp figure.pdf]
